# Perioperative Practice Patterns of Anaesthesiologists Surrounding Glucagon-Like Peptide-1 (GLP-1) Agonist Medications

**DOI:** 10.4274/TJAR.2025.241653

**Published:** 2025-03-21

**Authors:** Meghan Brennan, Sabrina H. Han, Kyle Ockerman, Sonia D. Mehta, Heather J. Furnas, Frederik Heath, Patricia Mars, Audrey Klenke, Sarah C. Sorice-Virk

**Affiliations:** 1 University of Florida College of Medicine, Department of Anaesthesiology, Gainesville, Florida; 2 University of Chicago, Department of Surgery, Division of Plastic and Reconstructive Surgery, Chicago, Illinois; 3 Stanford University, Department of Surgery, Division of Plastic and Reconstructive Surgery, Palo Alto, California; Plastic Surgery Associates &amp; Allegro MedSpa, Santa Rosa, California; 4 University of California Irvine School of Medicine, Irvine, California; 5 Venus by Mars Cosmetic Surgery Center, Tucson, Arizona; 6 Pinnacle Plastic Surgery, Beaufort, South Carolina; 7 Stanford University, Department of Surgery, Division of Plastic and Reconstructive Surgery, Palo Alto, California

**Keywords:** Anaesthesiologists, glucagon-like peptide-1, off-label use, physicians, practice patterns

## Abstract

**Objective:**

Aspiration of gastric contents during induction of anaesthesia is a rare but well-recognized complication with high morbidity and mortality risk. Patients at highest risk include those with full stomachs, diabetes, hiatal hernias, gastrointestinal obstructions, severe gastroesophageal reflux, and known delayed gastric emptying. Recently, the use of glucagon-like peptide-1 (GLP-1) agonists has expanded rapidly, including their application in cosmetic weight loss. This drug class suppresses glucagon release after meals, thereby delaying gastric emptying over an undefined duration. For patients taking these medications in the perioperative period, the effect on overall aspiration risk is unknown. This survey details the current practice pattern of anaesthesiologists regarding GLP-1 agonists.

**Methods:**

An IRB-approved 30-question, uncompensated survey was distributed to 30,096 self-reported actively practicing United States members of the American Society of Anesthesiologists (ASA). The survey collected demographic information, practice information, and included questions about the management of patients taking GLP-1 agonists. To ensure participant confidentiality, no identifiable information was collected.

**Results:**

The survey response rate was 5.98%, with 1,801 surveys returned. Ninety-seven percent of respondents indicated familiarity with GLP-1 agonists, and eighty-one percent indicated they had not personally witnessed complications in patients taking GLP-1 agonists. Most respondents indicated perioperative aspiration as the largest concern and the most commonly reported witnessed complication. 62% reported having “some” to “a lot” of experience providing anaesthesia to patients taking these medications. Most respondents reported NPO guidelines consistent with current ASA practice guidelines.

**Conclusion:**

The majority of anaesthesiologists report perioperative aspiration as their highest concern for patients taking this class of medications.

**Main Points**

• A nationwide survey of anaesthesiologists indicated their main concern for patients taking glucagon-like peptide-1 agonists, is increased risk of delayed gastric emptying and subsequent increased risk of aspiration on induction of general anaesthesia.

## Introduction

Management of type 2 diabetes (T2DM) and obesity has changed dramatically with the increased use of glucagon-like peptide-1 (GLP-1) agonists.^1, 2, 3, 4, 5, 6, 7, 8, 9^ Originally approved for the treatment of T2DM, GLP-1 agonists were found to have profound effects on weight loss, surpassing what was previously achievable with medication management alone (average <5% reduction in weight).^5^ Currently, semaglutide [brand name Ozempic (Novo Nordisk, Bagsværd, Denmark)] and tirzepatide [brand name Mounjaro (Eli Lilly and Company, Indianapolis, IN)] hold Food and Drug Administration (FDA) approval limited to the treatment of T2DM.^2, 4^ However, Wegovy (Novo Nordisk, Bagsværd, Denmark), a “sister” semaglutide, and Zepbound (Eli Lilly and Company, Indianapolis, IN), a “sister” tirzepatide, gained FDA approval for medical management of obesity in 2021 and 2023, respectively.^6, 7, 8^ In the past five years, these medications have increased in popularity, greatly.^10^ Additionally, a rising number of app-based weight loss programs with questionable screening and patient follow-up have raised patient safety concerns with the FDA and other governing bodies.^5^

GLP-1 agonists depress appetite by delaying gastric emptying and suppressing post-prandial glucagon release. Because this class of drugs has a longer half-life than endogenous GLP-1, the durations of these side effects related to long half-life and its weekly dosing regimen is uncertain. Given the potential for gastroparesis and retained gastric contents, anaesthesiologists are justified in their concerns about the significant risk of perioperative regurgitation and pulmonary aspiration syndrome, even when patients follow standard preoperative fasting times.^7^ The American Society of Anesthesiologists (ASA) issued a consensus statement that advocated discontinuing weekly-dosed GLP-1 medications 1 week prior to surgery. In cases where discontinuation of pre-operative GLP-1 agonists is not possible or if gastrointestinal symptoms (e.g., nausea, vomiting) are present, the ASA recommends a gastric ultrasound to assess stomach contents or proceeding with rapid sequence intubation (RSI) at induction.

Given the relative youth of these compounds and rapidly expanding indications, there are few published data on their utilization, anaesthetic implications, and subsequent perioperative management. In a recent letter to the anaesthesiology editor, Ushakumari and Sladen^12^ expressed that the current ASA guidelines lack the evidence to support them; however, the European Society of Anaesthesiology and Intensive Care (ESAIC) has recently updated guidelines with further evidence for preoperative considerations, for anaesthesiologists.^11, 12, 13^ To better understand how anaesthesiologists are navigating the ubiquitous use of GLP-1 agonists, this survey aims to explore their current practice patterns. As the use of GLP-1 agonists increases, this study seeks to enhance understanding of GLP-1 agonists’ effects on perioperative physiology and contribute to the development of evidence-based guidelines for safer anaesthetic management.

## Methods

### Survey Design

Institutional Review Board University of Florida exemption was obtained (approval no.: IRB202301912, date: 21.06.2024). The survey was based on a review of existing literature and included a pilot survey of 76 anaesthesiologists at a single academic tertiary care center. The study aimed to investigate current anaesthetic practice patterns and familiarity with GLP-1 agonists among practicing anaesthesiologists in the United States. The 30-question electronic survey included questions about gender, age range, race, years in practice, fellowship training, practice demographic and geographic area, anaesthesia-specific preoperative clinic status, and existing perioperative institutional guidelines surrounding GLP-1 agonists. Primary outcomes assessment was based on self-reported familiarity with GLP-1 agonists and experience and comfort providing anaesthesia to patients taking them. Respondents were also asked about complications, adverse events, management, NPO guidelines, and intubation strategy for patients taking GLP-1 agonists. No identifiable information was collected.

### Participant Selection

This uncompensated survey was disseminated to all actively practicing members in the United States of the ASA. The survey was sent out twice via email, with a two-week period between the initial survey email and the follow-up email.

### Data Analysis

Incomplete survey responses, as well as responses from those who indicated they were “Retired”, were excluded from analysis. Responses that were left blank were not included in the descriptive outcomes or analyses. The practice demographics were simplified to describe primarily inpatient or outpatient practice. Those who reported 50% or more outpatient practice were classified as having a primary focus on outpatient practice, with the others classified as having a primary focus on inpatient practice. Respondents who reported “some” or “a lot” of comfort with GLP-1 agonists were identified as “more experienced,” and the others were described as “less experienced.”

### Statistical Analysis

Statistical analysis was conducted using JMP Pro, Version 15 (SAS Institute Inc., Cary, NC). Responses to all questions were summarized. Chi-square tests were used to assess associations between demographic and clinical data and outcome responses. An alpha level of 0.05 was used for significance for all tests.

## Results

### Overall

The survey was sent to 30,096 actively practicing anaesthesiologists in the United States. 18,234 of the emails were opened, and 1,801 responses were received. This resulted in a response rate of 5.98% of the total and 9.9% from those who opened the email. Of those responses, two reported they were retired, and their responses were withheld from study analysis. Twenty-seven surveys were started, but the majority of responses were left blank and were removed from the study analysis as well, resulting in 1,772 responses being included in the analysis. Responses that were left blank for individual questions were not included in the study analysis.

### Demographics

Complete demographic data from the survey respondents are shown in Supplementary Table 1. Sixty-three percent of respondents were male, and 72% reported they identified as White or Caucasian. Sixty-three percent were less than 55 years old, and fifty-four percent reported they had been in practice for less than 10 years. Twenty-eight percent of respondents (496/1764) reported they worked in the Southeast region (AL, AR, FL, GA, KY, LA, MS, NC, SC, TN, VA, WV); 22% in the Midwest (IA, IL, IN, KS, MI, MN, MO, ND, NE, OH, SD, WI), and 21% in the Northeast (CT, DE, MA, MD, ME, NH, NJ, NY, PA, RI, VT). Over half of respondents reported working in an urban, (28%, 487/1759) or a major metropolitan (32%, 559/1759) area. Forty-one percent (726/1768) reported they had completed fellowship training. Fifty-eight percent (1031/1765) reported they worked in a private or group-owned practice. The majority reported they supervised residents or midlevel providers (78%, 1378/1761). Thirty-six percent of respondents (631/1767) reported a 50/50 split between inpatient and outpatient practice. Few (3%, 70/1767) reported they worked at a 100% inpatient practice, while 12% reported a 100% outpatient surgery practice. Twenty-six percent (454/1761) of respondents indicated their patients were evaluated in a preoperative anaesthesia clinic 100% of the time; 20%, (355/1761) reported their patients were never evaluated in a preoperative anaesthesia clinic.

### Patient Populations

Respondents indicated that diabetes was the most common indication (59%, 903/1532) for using GLP-1 agonists, followed by obesity management (23%, 350/1532). Ten percent, (151/1532) reported they were unaware of the prescription indication, and 8% (128/1532) reported a primary indication of cosmetic weight loss. Regarding the management of bridging medications in the perioperative period, 61% (261/426) of patients were managed by primary care providers, 30% (128/426) by the endocrinologist, and 6% (21/426) by the surgeon. Further details are shown in Table 1.

### NPO Guidelines/Practice Patterns

Most respondents (75%, 1066/1418) indicated their NPO guidelines for patients taking GLP-1 agonists did not differ from the standard ASA preoperative NPO guidelines for all patients. Five percent (72/1418) reported that patients were required to be NPO for 24 hours prior to surgery, and 3% (44/1418) indicated patients were excluded from “enhanced recovery after surgery” drinks. When asked if they altered intubation strategy for these patients, 39% of respondents (558/1418) reported using RSI or RSI with nasogastric tube suctioning, 15% (213/1418) reported altered intubation strategy by excluding laryngeal mask airway use, and 40% (563/1418) reported no alteration in intubation strategy.

Eighty-eight percent of respondents (1210/1383) reported that the type of surgery did not alter the duration patients were required to hold GLP-1 agonists prior to the day of the procedure. Eighty-seven percent of respondents (1208/1385) indicated that medication management for GLP-1 agonists did not differ between patients undergoing planned general anaesthesia and monitored anaesthesia care. For those patients taking GLP-1 agonists for primary diabetes management, 43% (612/1430) of respondents indicated they did not require their patients to hold the medication prior to undergoing elective surgery, 16% (224/1430) required holding for less than 1 week, 15% (214/1430) deferred to the primary care physician or endocrinologist, and 12% (180/1430) required holding the medication for 1 to 2 weeks. For those patients taking GLP-1 agonists for non-diabetic indications, 39% (545/1415) continued taking GLP-1 agonists before elective surgery, 18% (256/1415) held the medications between 1 and 2 weeks, and 15% (219/1415) held the medications for less than 1 week. Further details are shown in Table 2.

### Outcomes and Complications

Ninety-seven percent (1703/1759) of respondents answered that they were familiar with GLP-1 agonists. Sixty-two percent (1100/1767) answered that they had “some” to “a lot” of experience providing anaesthesia to patients taking GLP-1 agonists. Twenty-seven percent (406/1532) answered that they were extremely comfortable providing anaesthesia to patients taking GLP-1 agonists, while 16% reported they were “somewhat uncomfortable” or “extremely uncomfortable” most respondents (81%, 1243/1532) had not personally witnessed any perioperative complications in patients taking GLP-1 agonists. Aspiration was the most common complication reported (57%, 165/289) by those who witnessed perioperative complications. For those who had not personally witnessed a perioperative complication, the majority, (60%, 1001/1674) reported their biggest concern was related to higher perioperative aspiration risk from delayed gastric emptying. Further details are shown in Table 3.

### Comments From Respondents

Respondent comments regarding the need for increased guidance and education, aspiration concerns, uses and opinions on gastric ultrasound, airway management, and coordination of care concerns are shown in Table 4.

### Analyses

### Experience with GLP-1 agonists

To evaluate if there was any association between the demographic and clinical variables collected and those who reported “some” or “a lot” of comfort with GLP-1 agonists, experience with GLP-1 agonists was condensed from 4 categories into 2 categories: those who reported “some” or “a lot” of experience with GLP-1 agonists and those who reported “a little” or “none.”

A significant association was found between experience with GLP-1 agonists and the proportion of patients evaluated at a preoperative anaesthesia clinic (χ²: 11.1,* P*=0.0255). Those who reported their patients were evaluated at a preoperative anaesthesia clinic 75% to 100% of the time were 1.38 times more likely to report “some” or “a lot” of experience with GLP-1 agonists than those who reported <25% of patients were seen in a preoperative anaesthesia clinic; they were also 1.52 times more likely [Odds ratio (OR) 1.52 (1.12, 2.08), *P *value 0.0081] to report “some” or “a lot” of experience than those who reported their patients were seen 25% to 50% of the time in a preoperative anaesthesia clinic. Those who reported their patients were never seen in a preoperative anaesthesia clinic, were 1.48 times more likely [OR 1.48 (1.07, 2.06), *P *value 0.0184] to report “some” or “a lot” of experience with GLP-1 agonists than those who reported their patients were seen in a preoperative anaesthesia clinic 25% to 50% of the time.

Overall, there was no association between experience with GLP-1 agonists and changes to intubation strategy. However, when the data were examined across practice types, we found that those in private practice or group-owned practice who reported “some” or “a lot” of experience with GLP-1 agonists were 1.44 times more likely [OR 1.44 (1.06, 1.95), *P *value 0.0195] to alter their intubation strategy than those who reported “little” or “no” experience with GLP-1 agonists.

### Comfort with GLP-1 agonists

A significant association was found between comfort with GLP-1 agonists and the proportion of patients being seen at a preoperative anaesthesia clinic (χ² 11.71, *P *value 0.0197). Those who reported that patients are seen in a preoperative anaesthesia clinic 75% to 100% of the time were (1) 1.54 times more likely [OR 1.54 (1.01, 2.12), *P *value 0.0115] to report “some” or “a lot” of comfort with GLP-1 agonists than those who reported their patients are seen 25% to 50% of the time; (2) 1.6 times more likely [OR 1.6 (1.18, 2.17), *P *value 0.0026] to report “some” or “a lot” of comfort with GLP-1 agonists than those who reported their patients are seen 50% to 75% of the time in a preoperative anaesthesia clinic; and (3) 1.41 times more likely [OR 1.41 (1.04, 1.91), *P *value 0.0262] to report “some” or “a lot of” comfort than those who reported their patients are never seen in a preoperative anaesthesia clinic.

### Complications related to GLP-1 agonists

A significant association was found between those who reported witnessing complications in patients taking GLP-1 agonists and their experience level with GLP-1 agonists (χ² 30.65,* P* < 0.0001). Those who reported “some” or “a lot” of experience with GLP-1 agonists were 2.38 times more likely [OR 2.38 (1.72, 3.30), *P* < 0.0001] to report having witnessed complications in patients taking GLP-1 agonists. There was a significant association found between comfort level with GLP-1 agonists and witnessing complications in patients using these drugs: those who reported less comfort with GLP-1 agonists were 1.41 times more likely [OR 1.41 (1.09, 1.82), *P *value 0.0094] to report having witnessed complications in their use.

There was no association found between those who reported witnessing complications in patients taking GLP-1 agonists and practice demographic (>50% outpatient vs. inpatient) (χ² 1.04,* P *value 0.3069). No association was found between witnessing complications in patients taking GLP-1 agonists and geographic location (urban vs. suburban) (χ²: 0.48, *P *value 0.4899).

### Altered Intubation Strategy Related to GLP-1 Agonists

No significant association was found between experience level with GLP-1 agonists and alterations in intubation strategy in patients on such treatment (chi-square 2.83, *P *value 0.0927). Those who reported less comfort with GLP-1 agonists were 1.65 times more likely [OR 1.65 (1.33, 2.06), *P *value < 0.0001] to report altering their intubation strategy than those who reported “some” or “a lot” of comfort with GLP-1 agonists.

Those who reported having witnessed complications in patients taking GLP-1 agonists were 3.22 times more likely [OR 3.22 (2.34, 4.44), *P *value < 0.0001] to report they alter their intubation strategy than those who have not witnessed complications in these patients.

### Different NPO Guidelines for GLP-1 Agonists

A significant association was found between practice type and alterations in NPO guidelines for those taking GLP-1 agonists. Those who reported being in a private or group-owned practice were 1.53 times more likely [OR 1.53 (1.13, 2.09), *P *value 0.0067] to report using different NPO guidelines for patients taking GLP-1 agonists (compared to ASA standard guidelines) than those who reported being in academic practice. Similarly, they were 1.52 times more likely [OR 1.52 (1.07, 2.18), *P *value 0.0198] than those who reported being in hospital-employed practice. Of note, at the time of this survey, the ASA had not yet released its consensus statement and providers’ guidelines required longer NPO times than ASA standard guidelines.

## Discussion

GLP-1 agonists have soared in popularity due to their significant effects on weight loss. Our group previously demonstrated the exponential rise in internet search interest for “Ozempic,” “Wegovy,” and “Mounjaro” in a Google Trends analysis.^10^ In 2023, new GLP-1 agonist prescriptions for diabetes increased by 128% and prescriptions for obesity increased by 352%.^14^ The growing trend is likely to continue as researchers explore indications beyond diabetes and obesity for this drug class, including polycystic ovarian syndrome, Alzheimer’s disease, Parkinson’s disease, and nonalcoholic fatty liver disease.^15, 16, 17, 18, 19^ With increasing use, it is imperative to better understand how these medications can affect patients’ physiology in the perioperative setting.

Anaesthesiologists in our study indicated their highest concern for patients taking GLP-1 agonists, was increased aspiration risk on induction of anaesthesia, which was also the most witnessed complication. Delayed gastric emptying is most pronounced within the first 3 months of use, and may subside after 20 weeks according to one study.^20^ Other data on patients undergoing upper endoscopy suggest no predictability regarding the interval of GLP-1 agonists (e.g., semaglutide) discontinuation period and the prevalence of finding retained gastric contents at the time of endoscopy.^21^ In the setting of diabetes, patients already present with an elevated baseline risk of gastroparesis,^22^ and it is uncertain how GLP-1 agonists may further affect this risk.

The most recent ESAIC guidelines recommend to hold GLP-1 agonists at least one week, prior to scheduling procedures that require sedation or anaesthesia for patients who inject weekly.^11^ Furthermore, the guidelines specify that if the medications are given for obesity, then two weeks (i.e, three half-lives) are recommended. If the medication is prescribed as daily oral or subcutaneous administration, they recommend discontinuing GLP-1 agonists on the day of the procedure. Similarly, the current ASA consensus-based guidance statement on preoperative management of patients taking GLP-1 agonists advocates for patients undergoing elective procedures to hold these medications 1 week preoperatively and to evaluate patients for symptoms that could put them at increased risk of gastroparesis (e.g., nausea, bloating, or abdominal pain). If those symptoms are present, it is advised to treat patients as if they have a “full stomach” and to discuss these risks with the patient/surgical team or to consider delaying the procedure.^13^ However, from a pharmacologic standpoint, in order to avoid the aspiration risk induced by delayed gastric emptying, the medication would need to be held for at least 5 half-lives prior to surgery, and, depending on the specific GLP-1 agonist, could need to be held up to 2 or more weeks.^23^ This time interval raises practicality concerns, especially in settings without routine preoperative surgical, anaesthesia clinics. In the current study, only 26% of participants reported attending an anaesthesia preoperative clinic, while 20% reported attending none. Preoperative anaesthesia clinics have been shown to reduce patient morbidity and mortality.^24^ Therefore, facilitating preoperative care with additional precautions (i.e., medication bridging, collaboration with primary care providers, additional clearance) may thereby avoid potential perioperative complications. Given the increasing use of GLP-1 agonists, earlier evaluation by an anaesthesiologist or preoperative-specific team is likely beneficial for aspiration risk reduction, and in the case of diabetics, for improved glycemic control for optimal surgical outcomes if bridging medications are needed.

Other safety concerns include the broader availability via app-based prescription and pharmacy services, as a result, several deaths related to compounded GLP-1 agonists have been reported.^25, 26^ Preoperative anaesthesia clinics would enable a degree of quality control at least prior to surgery.

This survey illustrates considerable variation in respondents’ comfort levels with patients taking GLP-1 agonists and perioperative management of this population. Less than one-third of respondents felt extremely comfortable providing anaesthesia to patients taking GLP-1 agonists, while 16% felt “somewhat uncomfortable” or “extremely uncomfortable.” Those who were less comfortable were 1.65 times more likely to report altering their intubation strategy for patients taking these medications (*P* < 0.0001) and 1.41 times more likely to report witnessing complications (*P*=0.0094). While these findings do not demonstrate causality, a general sense of apprehension regarding the perioperative management of this patient population is evident, in conjunction with other findings of this and other studies. Anaesthesiologists who have witnessed complications are 3.22 times more likely, to report altering their intubation strategy than those who have not (*P *< 0.0001). Respondents in a private or group-owned practice setting with more experience with GLP-1 agonists were 1.44 times more likely to report altering their intubation strategy compared to those with little experience. Similarly, anesthesiologists who practice in a private or group-owned practice setting were 1.53 times more likely to report using different NPO guidelines for patients on GLP-1 agonists (compared to ASA standard guidelines).

Education for physicians and physician extenders in providing anaesthesia to patients taking these drugs is imperative. The ASA has indicated that increased research is needed to elucidate the effects of GLP-1 agonists on gastric emptying and aspiration risk. Our group is currently conducting a prospective study using ultrasonography to investigate gastric contents in the preoperative setting.

### Study Limitations

Our survey provides new insight into the perioperative practice patterns of anesthesiologists in patients taking GLP-1 agonists. However, there are several limitations. Overall, the response rate was low. Only 5.98% of those listed as actively practicing United States members who are ASA members responded to the survey. Therefore, the results of this survey may not be representative of the true population. Several questions required subjective assessments as well as self-reporting. The ASA has announced consensus-based guidance for preoperative management of patients taking GLP-1 receptor agonists since the survey was completed, those who requested increased guidance from the ASA may be satisfied with this statement.

## Conclusion

A primary concern held by the ASA members surveyed was that patients taking GLP-1 agonists may have delayed gastric emptying and subsequently an increased aspiration risk. Moreover, free-text responses indicated providers wanted further guidance from the ASA, as current guidelines may be insufficient regarding the NPO as well as medication cessation recommendations. As the use of these drugs ubiquitous, widespread implementation of preoperative anaesthesia clinics should be considered, and excellent communication with the surgical team is essential. Further mechanistic research in the perioperative setting is needed.

## Ethics

**Ethics Committee Approval:** Institutional Review Board University of Florida exemption was obtained (approval no.: IRB202301912, date: 21.06.2024).

**Informed Consent:** Survey study.

## Supplementary Materials

Supplementary Table 1. Demographic Data Click here to access: https://l24.im/bWX8

## Figures and Tables

**Table 1. Patient Population/Practice Information:** 

**Percent of time patients are evaluated at a preoperative anaesthesia clinic**	**n**	**% of total**
<25% of the time	332	18.85%
25-50% of the time	265	15.05%
50-75% of the time	355	20.16%
75-100% of the time	454	25.78%
Never	355	20.16%
**Supervision of midlevel providers (residents, nurse anaesthetists, anaesthesia assistants)**	**n**	**% of total**
No	383	21.75%
Yes	1378	78.25%
**Average BMI in practice region**	**n**	**% of total**
<25	21	1.20%
>40	29	1.66%
25-30	219	12.53%
30-35	1021	58.41%
35-40	458	26.20%
**BMI cut-off for elective surgical procedures**	**n**	**% of total**
No BMI cut-off	1360	77.36%
Yes, <30	3	0.17%
Yes, <35	8	0.46%
Yes, <40	56	3.19%
Yes, <45	102	5.80%
Yes, <50	229	13.03%
**Familiarity with a class of drugs called GLP-1 agonists (i.e., Ozempic, Wegovy, Mounjaro)**	**n**	**% of total**
No	56	3.18%
Yes	1703	96.82%
**Experience level providing anaesthesia to patients who take GLP-1 agonists**	**n**	**% of total**
No	219	12.39%
Yes, a little	448	25.35%
Yes, a lot	296	16.75%
Yes, some	804	45.50%
**Comfort level with providing anaesthesia to patients taking GLP-1 medications**	**n**	**% of total**
Extremely comfortable	406	26.50%
Extremely uncomfortable	21	1.37%
Neither comfortable nor uncomfortable	391	25.52%
Somewhat comfortable	496	32.38%
Somewhat uncomfortable	218	14.23%
**Most common indication for which patients have been prescribed a GLP-1 agonist**	**n**	**% of total**
Cosmetic weight loss	128	8.36%
Primary diabetes management	903	58.94%
Primary obesity management	350	22.85%
Unknown	151	9.86%

BMI, body mass index; GLP-1, glucose-dependent insulinotropic peptide.

**Table 2. NPO Guidelines/Practice Patterns:** 

**In patients taking GLP-1 medication for DIABETIC indications, how long do you advise patients to hold GLP-1 medications prior to undergoing elective surgery?**	**n**	**% of total**
<1 week	224	15.66%
>2 weeks	40	2.80%
1-2 weeks	180	12.59%
Defer to PCP or endocrinologist	214	14.97%
Defer to surgeon	39	2.73%
I do not have them hold it	612	42.80%
I temporarily decrease the dosage	4	0.28%
Other	117	8.18%
**In patients taking GLP-1 medication for DIABETIC indications with HELD medications, do you temporarily transition the patient to another medication? **	**n**	**% of total**
No	953	62.29%
Yes, defer to other provider	467	30.52%
Yes, other	18	1.18%
Yes, transition to insulin	92	6.01%
**Management of bridging medications**	**n**	**% of total**
Endocrinologist	128	30.05%
Other	16	3.76%
PCP	261	61.27%
Surgeon	21	4.93%
**In patients taking GLP-1 medication for NON-DIABETIC indications, how long do you advise patients to hold GLP-1 medications prior to undergoing elective surgery? **	**n**	**% of total**
<1 week	219	15.48%
>2 weeks	92	6.50%
1-2 weeks	256	18.09%
>3 Weeks	7	0.49%
>4 weeks	12	0.85%
Defer to PCP or endocrinologist	141	9.96%
Defer to surgeon	48	3.39%
I do not have them hold it	545	38.52%
I temporarily decrease the dosage	5	0.35%
Other	90	6.36%
Does the duration of how long you recommend patients hold the medication differ whether the anaesthesia plan is monitored anaesthesia care versus general anaesthesia?	n	% of total
No	1208	87.22%
Yes	177	12.78%
Does the duration of how long you recommend patients hold the medication differ based on surgery type?	n	% of total
No	1210	87.49%
Yes	173	12.51%
Does the duration of how long you recommend patients hold the medication differ whether the patient will be admitted or be discharged home the day of surgery?	n	% of total
No	1315	95.29%
Yes	65	4.71%
Do your NPO guidelines differ from the recommended ASA guidelines for patients taking this medication?	n	% of total
No	1066	75.18%
Other	103	7.26%
Yes, they are excluded from ERAS drinks	44	3.10%
Yes, they are required to be NPO for 12 hours prior to surgery	133	9.38%
Yes, they are required to be NPO for 24 hours prior to surgery	72	5.08%
Do you alter your intubation strategy for patients who have been taking GLP-1 agonists?	n	% of total
No	563	39.70%
Other	84	5.92%
Yes, excludes patient as LMA candidate	213	15.02%
Yes, rapid sequence intubation	427	30.11%
Yes, rapid sequence intubation and nasogastric tube to suction	131	9.24%
Does the duration of how long you recommend patients hold the medication differ whether the anaesthesia plan is monitored anaesthesia care versus general anaesthesia?	n	% of total
No	1208	87.22%
Yes	177	12.78%
**Does the duration of how long you recommend patients hold the medication differ based on surgery type?**	**n**	**% of total**
No	1210	87.49%
Yes	173	12.51%
**Does the duration of how long you recommend patients hold the medication differ whether the patient will be admitted or be discharged home the day of surgery?**	**n**	**% of total**
No	1315	95.29%
Yes	65	4.71%
**Do your NPO guidelines differ from the recommended ASA guidelines for patients taking this medication?**	**n**	**% of total**
No	1066	75.18%
Other	103	7.26%
Yes, they are excluded from ERAS drinks	44	3.10%
Yes, they are required to be NPO for 12 hours prior to surgery	133	9.38%
Yes, they are required to be NPO for 24 hours prior to surgery	72	5.08%
**Do you alter your intubation strategy for patients who have been taking GLP-1 agonists?**	**n**	**% of total**
No	563	39.70%
Other	84	5.92%
Yes, excludes patient as LMA candidate	213	15.02%
Yes, rapid sequence intubation	427	30.11%
Yes, rapid sequence intubation and nasogastric tube to suction	131	9.24%
**Is management affected by surgery type**	**n**	**% of total**
No	1210	87.49%
Yes	173	12.51%
**Is management affected by postoperative discharge plans**	**n**	**% of total**
No	1315	95.29%
Yes	65	4.71%

ASA, American Society of Anesthesiologists; ERAS, enhanced recovery after surgery; GLP-1, glucose-dependent insulinotropic peptide; LMA, laryngeal mask airway; PCP, primary care provider.

**Table 3. GLP-1 Complications:** 

**What is the most common indication for which patients have been prescribed a GLP-1 agonist?**	**n**	**% of total**
Cosmetic weight loss	128	8.36%
Primary diabetes management	903	58.94%
Primary obesity management	350	22.85%
Unknown	151	9.86%
**Personally witnessed complications**	**n**	**% of total**
None	1243	81.14%
Perioperative aspiration	165	11%
Perioperative ketoacidosis or non-diabetic normoglycemic ketoacidosis	51	3%
Other	92	6%
**Highest perceived complication risk**	**n**	**% of total**
Higher perioperative aspiration risk from delayed gastric emptying	1370	82%
Perioperative ketoacidosis or non-diabetic normoglycemic ketoacidosis	450	27%
Other	199	12%
None	30	2%

GLP-1, glucose-dependent insulinotropic peptide.

**Table 4. Survey Responses:** 

**Need for increased guidance/education from the ASA**	I am very concerned about this class of medications and delayed gastric emptying. We have no ability at my center to advise patients to alter their medications other than [on the] day of surgery. I am interested in an official ASA guideline on this so we can circulate to surgeons to be considered when scheduling patients at our ASC.
-	Very interested in updated practice guidelines. We are at a constant battle with GI physicians for outpatient endoscopy and patients taking this medicine for weight loss. Help from ASA would be greatly appreciated.
-	Intubation strategy changes and NPO guidance is needed. Also, a strong recommendation for ERAS exemption is also needed given the observed gastric emptying delays.
-	I am very interested to see what the ASA has to say about this drug and potential statements. My group would be willing to change NPO status/induction management and work with our preoperative clinic for these patients.
-	I am acutely aware of this concern and have alerted our providers to watch for this class of medications and consider altering practice. We have not recommended holding these drugs preoperatively at this point, but will consider this going forward.
-	Many concerns regarding aspiration risk especially when NPO for minimum recommended time per ASA guidelines. Would like more direction on gastric emptying, aspiration risk, and if [there is a] difference between patients taking for DM vs weight loss.
-	We recently initiated changes to include a two-week discontinuation of these medications because of increased aspiration risk. We primarily do sedation anaesthesia for cosmetic procedures, and I do not feel comfortable sedating patients that have been on this medication because of the possibility of having a full stomach despite the NPO normal guidelines.
-	I would like to see guidelines for all types of anesthetics especially for outpatient endoscopies (EGD and colonoscopy). We need to have a guideline to show GI docs specifically the requirement, otherwise I am very concerned for increased aspiration frequency.
**Aspiration concerns**	I have colleagues with a GI practice that see significant retained gastric contents on patients on GLP-1 agonists. I strongly feel their aspiration risk is increased, so thank you for trying to get clarity on proper management.
-	We are hearing anecdotal reports of fasted EGD patients with undigested food seen in their stomachs. Anxiously awaiting some specialty specific guidelines, and in the meantime, taking a case by case conservative approach.
-	I see stomachs full of food in EGDs in patients on these drugs. Disaster waiting to happen. For obesity treatment should be stopped preop[eratively]. Diabetics have gastroparesis anyway, so maybe they are not tolerating these as well; I don’t see as many people on it just for diabetes.
-	From what I’ve read, the delayed gastric emptying is a concern but not consistent; severe aspiration has been rare but those instances are concerning to me - would be great if the ASA came out with a practice advisory.
-	Wondering if these patients should be excluded from a MAC case or even an LMA. Aspiration is obviously a major complication but definitely one we want to avoid at a stand alone surgery center.
**Gastric ultrasound uses/options**	I typically gastric ultrasound patients prior to the OR to assess gastric volume.
-	Would like to see a study where point of care ultrasound (POCUS) is used to evaluate these patients for gastric contents under routine ASA fasting guidelines.
-	It would be interesting to see research either with residual stomach content or ultrasound for stomach volumes.
-	I extensively assess patients satiety, nausea or fullness. My goal is POCUS soon.
-	Would like to see a study where pocus is used to evaluate these patients for gastric contents under routine ASA fasting guidelines.
-	Most common instance we see is GI work-up for side effects related to medication (e.g., nausea). In these patients, our group is recommending 2 days [of] clears with 12 hours NPO. We are trying to schedule them at a facility with anaesthesia machines and ultrasound available. The thought is to do gastric ultrasound in pre op. Some partners are planning to intubate all of these patients due to prior “possible aspiration” events.
-	Little direction from ASA regarding appropriate practice; will occasionally use gastric ultrasound if curvalinear probe available at facilities; have heard of several incidences of delayed gastric emptying in the region- but none at our practice.
**Airway management**	Several institutions have increased the pre-surgical NPO time for solids for these patients (our institution has not yet), but the exact data is unknown. I know a few institutions that are working on clinical studies right now to further explore this issue. I don’t trust the patients to be NPO under the normal ASA guidelines if they have taken their medication in the past week, so I strongly prefer ETT + RSI for them over MAC or LMA.
-	My current management of patients taking GLP-1 agonists include intubation with RSI and exclude the use of LMAs. With reference to MAC cases, I make it a point of keeping the patients lighter and maintain upper airway reflexes/protection. I solidly believe that these patients should be treated as an increased aspiration risk just as we would for gastroparesis patients.
-	As a private practice anesthesiologist, we are seeing a spike in the number of patients taking these medications for weight loss and we are struggling how to manage these patients since there is very little guidance. We are now intubating patients that normally we would perform a TIVA or LMA’s on to reduce the risk of aspiration.
-	I do not have them hold it because of its long half life. I treat them as full stomach precautions. If MAC, I keep them much lighter. If GA, I will RSI and not use an LMA. I am very concerned about aspiration and very much waiting for some practice guidelines to standardize our practice surrounding these medications.
-	I have not yet modified my intubation strategy but I am very seriously considering doing RSI for all these patients.
-	I inform the patient that there may be a higher risk of aspiration and I am more likely to intubate a patient in cases that I would have used an LMA or avoided intubation when patients are on this medication.
-	To summarize, we currently have no policies in place changing our practice, though some individual providers have made some practice changes (as noted). None of my patients have had complications (that I know of), but some of my CRNAs have [had] first-hand issues at other institutions (e.g., aspiration). As the popularity of these drugs spread, we are facing a big problem, so thank you for doing this. I’m looking forward to your results.
**Coordination of care concerns**	We don’t have really any official guidelines for these medications right now at our institution, which is why we generally defer to the PCP for perioperative management. That being said, the PCP does not know of the emerging risks/issues we are seeing with these patients in the perioperative period. In discussions with colleagues, we have SO MANY experiences with seeing delayed gastric emptying with these patients.
-	Glad to see someone is working on guidelines for this population. Not only anesthesiologist, but surgeons and PCPs seem to be unaware of the risks associated to these medications. They are still been managed as any other patient and included in ERAS protocols. Very worrisome and dangerous.
-	Hopefully the ASA can partner with endocrine societies for some formal recommendations.

ASA, American Society of Anesthesiologists; ASC, ambulatory surgery center; CRNAs, certified registered nurse anesthetists; EGD, esophagogastroduodenoscopy; ERAS, enhanced recovery after surgery; ETT, endotracheal tube; GA, general anaesthesia; GI, gastrointestinal; GLP-1, glucagon-like peptide-1; LMA, laryngeal mask airway; MAC, monitored anaesthesia care; NPO, nil per os; OR, operating room; PCP, primary care provider; POCUS, point-of-care ultrasound; RSI, rapid sequence intubation; TIVA, total intravenous anaesthesia.
