# Erratum

**DOI:** 10.4274/TJAR.2025.e001

**Published:** 2025-05-30

“Brennan M, Han SH, Ockerman K, Mehta SD, Furnas HJ, Heath F, Mars P, Klenke A, Sorice-Virk SC. Perioperative Practice Patterns of Anaesthesiologists Surrounding Glucagon-Like Peptide-1 (GLP-1) Agonist Medications. Turk J Anaesthesiol Reanim. 2025 Apr;53(2):42-52. doi:10.4274/TJAR.2025.241653.”

Acknowledgements information has been added before the Footnotes heading on page 51.

**Published on page 51;**

**Ethics**

**Ethics Committee Approval:** Institutional Review Board University of Florida exemption was obtained (approval no.: IRB202301912, date: 21.06.2024).

**Informed Consent:** Survey study.

**Footnotes**

**Author Contributions:** Surgical and Medical Practices - S.C.S.-V., M.B., S.H.H.,; Concept - S.C.S.-V., M.B., S.H.H., A.K., P.M., S.D.M., H.J.F., F.H.; Design - S.C.S.-V., M.B., S.H.H., F.H.; Data Collection and/ or/Processing - S.C.S.-V., K.O., M.B., S.H.H., F.H.; Analysis and/or/ Interpretation - S.C.S.-V., K.O., M.B., S.H.H., F.H.; Literature Review - S.C.S.-V., K.O., M.B., S.H.H., F.H.; Writing - S.C.S.-V., K.O., M.B., S.H.H., F.H.

**Declaration of Interests:** The authors declare no conflicts of interest.

**Funding:** No funding was received for conducting this study.

**Corrected page 51;**

**(The added parts are given in italic font)**

**Ethics**

**Ethics Committee Approval:** Institutional Review Board University of Florida exemption was obtained (approval no.: IRB202301912, date: 21.06.2024).

**Informed Consent:** Survey study.

**Acknowledgments:** The authors thank Bryan Penberthy, MFA, of the University of Florida College of Medicine Department of Anesthesiology’s Communications & Publishing office for his editorial assistance with this manuscript.

**Footnotes**

**Author Contributions:** Surgical and Medical Practices - S.C.S.-V., M.B., S.H.H.,; Concept - S.C.S.-V., M.B., S.H.H., A.K., P.M., S.D.M., H.J.F., F.H.; Design - S.C.S.-V., M.B., S.H.H., F.H.; Data Collection and/ or/Processing - S.C.S.-V., K.O., M.B., S.H.H., F.H.; Analysis and/or/ Interpretation - S.C.S.-V., K.O., M.B., S.H.H., F.H.; Literature Review - S.C.S.-V., K.O., M.B., S.H.H., F.H.; Writing - S.C.S.-V., K.O., M.B., S.H.H., F.H.

**Declaration of Interests:** The authors declare no conflicts of interest.

**Funding:** No funding was received for conducting this study.

